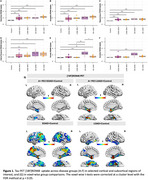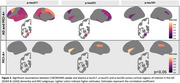# [^18^F]RO948 Tau PET Imaging in Early and Late‐Onset Alzheimer's Disease: Regional Evolution and Correlations with Plasma Biomarkers

**DOI:** 10.1002/alz70856_104754

**Published:** 2026-01-07

**Authors:** Mariola Zapater‐Fajari, Marco Bucci, Konstantinos Chiotis, Anders Wall, Jonas Eriksson, Gunnar Antoni, Ilaria Pola, Kübra TAN, Wiebke Traichel, Andrea Benedet, Nicholas Ashton, Kaj Blennow, Henrik Zetterberg, Nenad Bogdanovic, Agneta K Nordberg

**Affiliations:** ^1^ Department of Neurobiology, Care Sciences and Society, Division of Clinical Geriatrics, Center for Alzheimer Research, Karolinska Institutet, Stockholm, Sweden; ^2^ Turku PET Centre, Turku University Hospital, University of Turku and Åbo Akademi University, Turku, Finland; ^3^ Department of Neurology, Karolinska University Hospital, Stockholm, Sweden; ^4^ Department of Surgical Sciences, Section of Nuclear Medicine & PET, Uppsala University, Uppsala, Sweden; ^5^ Department of Medicinal Chemistry, Uppsala University, Uppsala, Sweden; ^6^ PET Centre, Uppsala University Hospital, Uppsala, Sweden; ^7^ Department of Psychiatry and Neurochemistry, Institute of Neuroscience and Physiology, The Sahlgrenska Academy, University of Gothenburg, Mölndal, Sweden; ^8^ King's College London, Institute of Psychiatry, Psychology & Neuroscience, Maurice Wohl Clinical Neuroscience Institute, London, United Kingdom; ^9^ NIHR Biomedical Research Centre for Mental Health and Biomedical Research Unit for Dementia at South London and Maudsley NHS Foundation, London, United Kingdom; ^10^ Clinical Neurochemistry Laboratory, Sahlgrenska University Hospital, Mölndal, Västra Götaland län, Sweden; ^11^ Department of Neurodegenerative Disease, UCL Institute of Neurology, London, United Kingdom; ^12^ Hong Kong Center for Neurodegenerative Diseases, Hong Kong, Hong Kong, China; ^13^ University of Wisconsin School of Medicine and Public Health, Madison, WI, USA; ^14^ Theme Inflammation and Aging, Karolinska University Hospital, Stockholm, Sweden

## Abstract

**Background:**

Using [18F]RO948 as a tau PET ligand, we assessed tau deposition in a cohort of memory clinic patients and cognitively unimpaired controls. Regional tau binding was then compared to levels of plasma *p*‐Tau217, *p*‐Tau181, *p*‐Tau231 biomarkers.

**Methods:**

Thirty‐seven patients from the Memory Clinic at Karolinska University Hospital Huddinge were evaluated: 27 patients with biomarker confirmed AD (CSF A+) at clinical stage of MCI (*n* = 14) or dementia (*n* = 13). Thirteen cases qualified for an early onset disease (EOAD) and fourteen for late onset (LOAD), and 10 cognitively normal (CN) participants. On the same day, participants underwent [^18^F]RO948 tau PET imaging, magnetic resonance imaging (MRI), and blood sampling for plasma biomarker analysis using the NuLISAseq (Alamarbio) CNS panel.

**Results:**

EOAD and LOAD patients, whether MCI or dementia, showed significantly higher [18F]RO948 uptake in the entorhinal cortex, hippocampus and amygdala compared to the CN participants (Figure A‐C). Only the EOAD dementia patients displayed significantly higher uptake in the parietal cortex compared to CN participants but also compared to the other AD subgroups (*p* = 0.04) (Figure 1E‐F). Voxel‐wise analyses showed a significantly higher [18F]RO948 uptake confined in medial temporal lobe areas in the patients with EOAD MCI or LOAD MCI compared to the CN. The LOAD dementia patients showed involvement primarily of the temporal neocortex while the EOAD dementia a broader involvement of even parietal and frontal cortices (Figure 1G). *p*‐Tau217, *p*‐Tau231, and *p*‐Tau181 plasma levels were elevated in both MCI and dementia patients, compared to controls (data not shown, all *p* < 0.05). In the dementia patients (EOAD and LOAD), plasma *p*‐Tau217 exhibited a stronger correlation with [18F]RO948 uptake across multiple brain regions compared to *p*‐Tau231 and *p*‐Tau181 where less broad areas were involved. In the MCI patients (EOAD and LOAD) *p*‐Tau217 correlated with uptake in the amygdala (Figure 2).

**Conclusions:**

[^18^F]RO948 PET can capture tau deposition at early stages of AD, particularly in medial temporal regions when plasma *p*‐Tau217 levels similarly elevated. As the disease progresses to the clinical dementia stage, tau accumulation extends into cortical regions, with distinct regional deposition patterns observed between EOAD and LOAD.